# Direct healthcare expenditure on Alzheimer’s disease from healthcare providers’ perspective in Malaysia: a micro-costing approach

**DOI:** 10.1038/s41598-024-69745-1

**Published:** 2024-08-14

**Authors:** Siew Chin Ong, Lyn Xuan Tay, Teck Fah Yee, Ewe Eow Teh, Alan Swee Hock Ch’ng, Rizah Mazzuin Razali, Wan Chieh Lim, Ungku Ahmad Ameen Bin Ungku Mohd. Zam, Thaigarajan Parumasivam

**Affiliations:** 1 Discipline of Social and Administrative Pharmacy, Universiti Sains Malaysia, 11800 Pulau Pinang, Malaysia; 2 Pharmacy Department, Hospital Queen Elizabeth, Ministry of Health Malaysia, 88586 Kota Kinabalu, Sabah Malaysia; 3 Department of Psychiatry & Mental Health, Hospital Pulau Pinang, Ministry of Health Malaysia, 10990 Pulau Pinang, Malaysia; 4 Department of Medicine, Seberang Jaya Hospital, Ministry of Health Malaysia, 13700 Seberang Perai, Penang Malaysia; 5 Geriatric Unit, Department of Medicine, Kuala Lumpur Hospital, Ministry of Health Malaysia, 50586 Kuala Lumpur, Malaysia; 6 Geriatrics Unit, Internal Medicine Department, Taiping General Hospital, Ministry of Health Malaysia, 34000 Taiping, Perak Malaysia; 7 Geriatrics Unit, Medical Department, Hospital Tengku Ampuan Rahimah Klang, Ministry of Health Malaysia, 41200 Klang, Selangor Malaysia; 8 Discipline of Pharmaceutical Technology, Universiti Sains Malaysia, 11800 Pulau Pinang, Malaysia

**Keywords:** Health care economics, Health policy, Health services, Public health, Outcomes research

## Abstract

Alzheimer’s disease (AD) is an important geriatric disease that creates challenges in health policy planning. There is no previous attempt to quantify the actual direct healthcare cost of AD among older adults in Malaysia. This retrospective observational study with bottom-up micro-costing approach aimed to evaluate the direct healthcare expenditure on AD along with its potential predictors from healthcare providers’ perspective, conducted across six tertiary hospitals in Malaysia. AD patients aged 65 and above who received AD treatment between 1 January 2016 and 31 December 2021 were included. Direct healthcare cost (DHC) of AD was estimated by extracting one-year follow-up information from patient medical records. As a result, 333 AD patients were included in the study. The mean DHC of AD was estimated RM2641.30 (USD 572.45) per patient per year (PPPY) from the healthcare payer’s perspective. Laboratory investigations accounted for 37.2% of total DHC, followed by clinic care (31.5%) and prescription medicine (24.9%). As disease severity increases, annual DHC increases from RM2459.04 (mild), RM 2642.27 (moderate), to RM3087.61 (severe) PPPY. Patients aged 81 and above recorded significantly higher annual DHC (p = 0.003). Such real-world estimates are important in assisting the process of formulating healthcare policies in geriatric care.

## Introduction

Dementia is a clinical syndrome under the domain of neurocognitive disorder that includes many etiologies such as Alzheimer’s dementia, vascular dementia, Lewy body dementia and others^1^. Being the major aetiology of dementia and more prevalent in older people, Alzheimer’s disease (AD) is recognised as a worldwide public health concern clinically, economically and socially^2,3^. According to the World Health Organisation (WHO), approximately 55 million individuals suffered from dementia in 2022, with AD being the major aetiology. This number is projected to rise significantly, reaching an estimated 132 million globally by 2050. Nearly two-thirds of this increase is expected to occur in low to middle-income countries (LMICs)^4^.

According to the United Nations, an ageing country is where the percentage of people aged 65 years and above reaches 7% of the total population^5^. Based on the current population estimates, Malaysia achieved an aging nation status in 2021 when the elderly population composition reached 7% and continued to increase to 7.4% in 2023^6^. In the near future, Malaysia will turn into an aged nation which 14% of the total population is estimated to reach 60 years and above in 2030. Such trend is projected to increase further in 2050 where one out of four residents in Malaysia is aged 60 and above^7^. In view of this, geriatric diseases including AD is gradually emerging as a healthcare concern in Malaysia due to the inevitable fact that Malaysia is experiencing population aging.

The worldwide cost of dementia was estimated at USD818 billion in 2015 and is estimated to reach USD2 trillion in 2030^8^. The cost of dementia in LMICs is substantial, but obtaining accurate estimates is challenging due to the lack of standardised cost assessment methods^4^. Many cost-of-illness studies specifically looking at AD were limited to high-income countries such as USA, UK, Spain, Sweden and Finland^4,9^, while few studies were conducted outside these Western regions. In Asia, dementia cost evaluations were conducted regardless of the etiology of this neurocognitive disorder^4^. It is estimated that dementia costs will reach USD92 million in 2030 and are further projected to reach USD185 million in 2050^10^. From several studies conducted in countries such as Singapore, Thailand, Philippines and Japan, the annual direct medical burden borne by the healthcare providers ranged from USD237.40 and USD3778.32 per patient with dementia^11–14^. Such estimates are useful in understanding the financial impact of the burden but lack specificity towards the etiology of diseases. In fact, etiology differences may result in different patterns of healthcare resource utilization that eventually reflect on healthcare providers’ expenditure. For instance, the annual medical cost of a patient with vascular dementia was found to be USD6797 higher than that of a patient with Alzheimer’s disease^15^. Such scenarios were also observed in Argentina, where both vascular and frontotemporal dementia incurred higher annual direct costs than AD due to a higher hospitalization rates in vascular dementia and higher psychotropic costs in frontotemporal dementia^16^. With that, it is clear that real-world evidence of country-specific estimates are very important to reflect the actual burden of a disease^17^. In 2015, a cross-sectional study conducted in Malaysia reported that direct healthcare cost (DHC) of dementia has a great influence on the disease burden from the healthcare provider’s perspective^18^. On average, RM10,034 was spent for each episode of dementia hospitalization in Malaysia^19^. Such cost figures were much higher than the average expenditure of community-dwelling older persons seeking healthcare in Malaysia (i.e., average of around RM426.50 annually)^20^. However, the study was looking at dementia patients generally regardless of the underlying cause.

Considering the scarcity of healthcare resources, healthcare stakeholders and policymakers require updated real-world cost estimates to aid their resource allocation decision-making process^21^. This is especially important in the scenario of rising healthcare utilization among older persons in the post-COVID era^22^. Furthermore, the scarcity of empirical evidence concerning the cost of AD in Malaysia underscores the importance of addressing this gap to comprehend the true disease burden. The current study endeavoured to distinguish AD, which is more prevalent in older people, from the general clinical syndrome of dementia. Therefore, this study aimed to evaluate the DHC, and the potential cost predictors incurred by older adults aged 65 years old and above affected by AD in Malaysia, employed a bottom-up micro-costing approach.

## Methodology

### Study design

This study was a retrospective observational study conducted in 6 public tertiary hospitals in Malaysia namely Pusat Jantung Sarawak (PJS), Hospital Pulau Pinang (HPP), Hospital Seberang Jaya (HSJ), Hospital Taiping (HTPG), Hospital Tengku Ampuan Rahimah (HTAR), and Hospital Kuala Lumpur (HKL). Bottom-up micro costing approach was employed in the current study. A standardized data collection form was designed to capture socio-demographics, medical history, AD severity, and health resource use. To categorise patients into different disease severity, the Diagnostic and Statistical Manual of Mental Disorders (DSM 5th Ed) evaluated by geriatricians and/or psychiatrists was employed by the principal and co-investigators on-site.

### Sampling criteria

**Figure 1:**
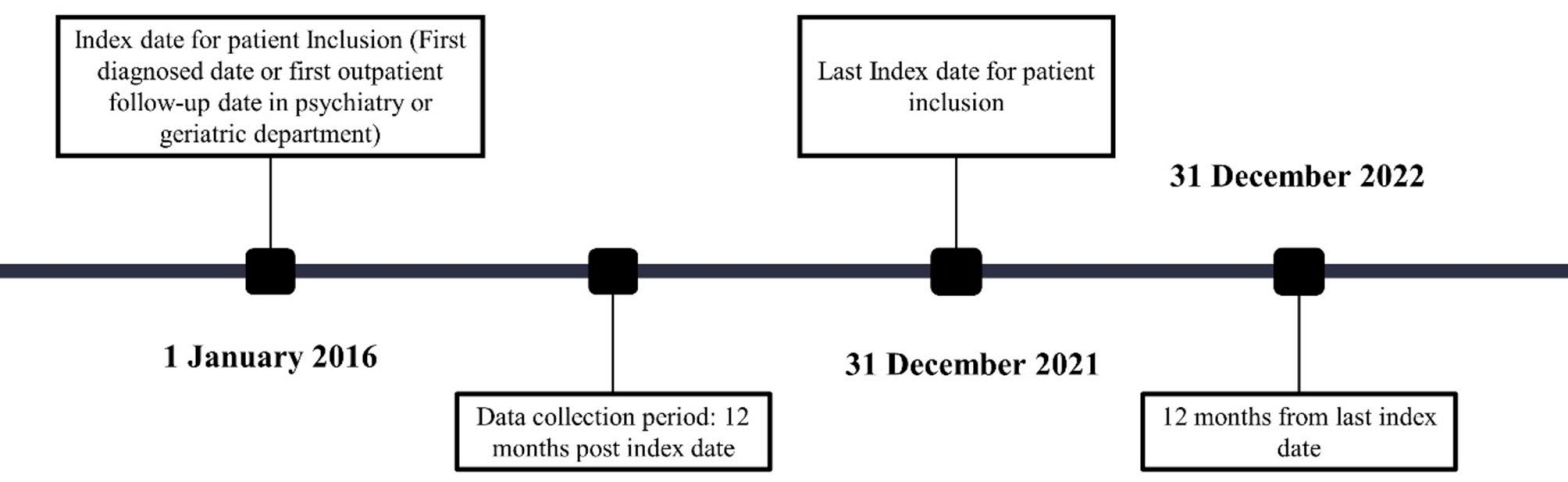
Data collection period of the study with index dates.

Eligible study population were patients aged 65 years old and above with a clinical diagnosis of AD (as documented in medical records) from 1 January 2016 to 31 December 2021. An index date was recorded for each input from the date of the first diagnosis of AD in the clinic or the date of the first presentation to the clinic with a diagnosis of AD. Retrospective data collection was done for up to one year as shown in Fig. 1. However, patients without regular follow up in the same hospital or those who participated in an interventional clinical trial in the post-index period were not included in the study.

### Sample size estimation

For the expected prevalence of 8.5% from National Health Morbidity Survey 2018 Elderly Health Volume 2^23^, the required sample size was 134 for the margin of error or absolute precision of ± 5% with 95% confidence and considering the potential loss/attrition of 10%. With this sample size, the anticipated 95% CI was (3.5%, 13.5%)^24^. Before the beginning of the study in knowing the finite population, a sample size of 300 with AD was determined. A total of 333 patient records which met the eligibility criteria for the study were obtained from the medical record unit at the designated study sites. Despite the actual sample size exceeding the initially estimated figure, this deviation was deemed suitable given the retrospective nature of the data collection. Importantly, the data collection process was conducted without direct patient involvement.

### Data collection and outcome measured

The primary outcomes measured in the study is the 1-year resource utilization and direct healthcare costs (DHC) associated with AD, commencing from the index date. As the current study focused on the healthcare provider's perspective, out-of-pocket expenses borne by patients including private healthcare costs were not considered during the data collection process. Out-of-pocket expenditures are one of the cost components for studies looking from the societal perspective. The utilization of healthcare resources at study sites, including expenses related to diagnostic assessments, outpatient visits, inpatient admissions, laboratory investigations, community healthcare services, and medications were recorded. The data on resource utilization and healthcare costs associated with AD were sought on an individual basis. Furthermore, the cost estimates were stratified based on the severity of the disease. The Diagnostic and Statistical Manual of Mental Disorders (DSM 5th Ed) was employed by the principal and co-investigators on-site to classify disease severity documentation in the medical records^1^.

### Costing methodology

**Figure 2:**
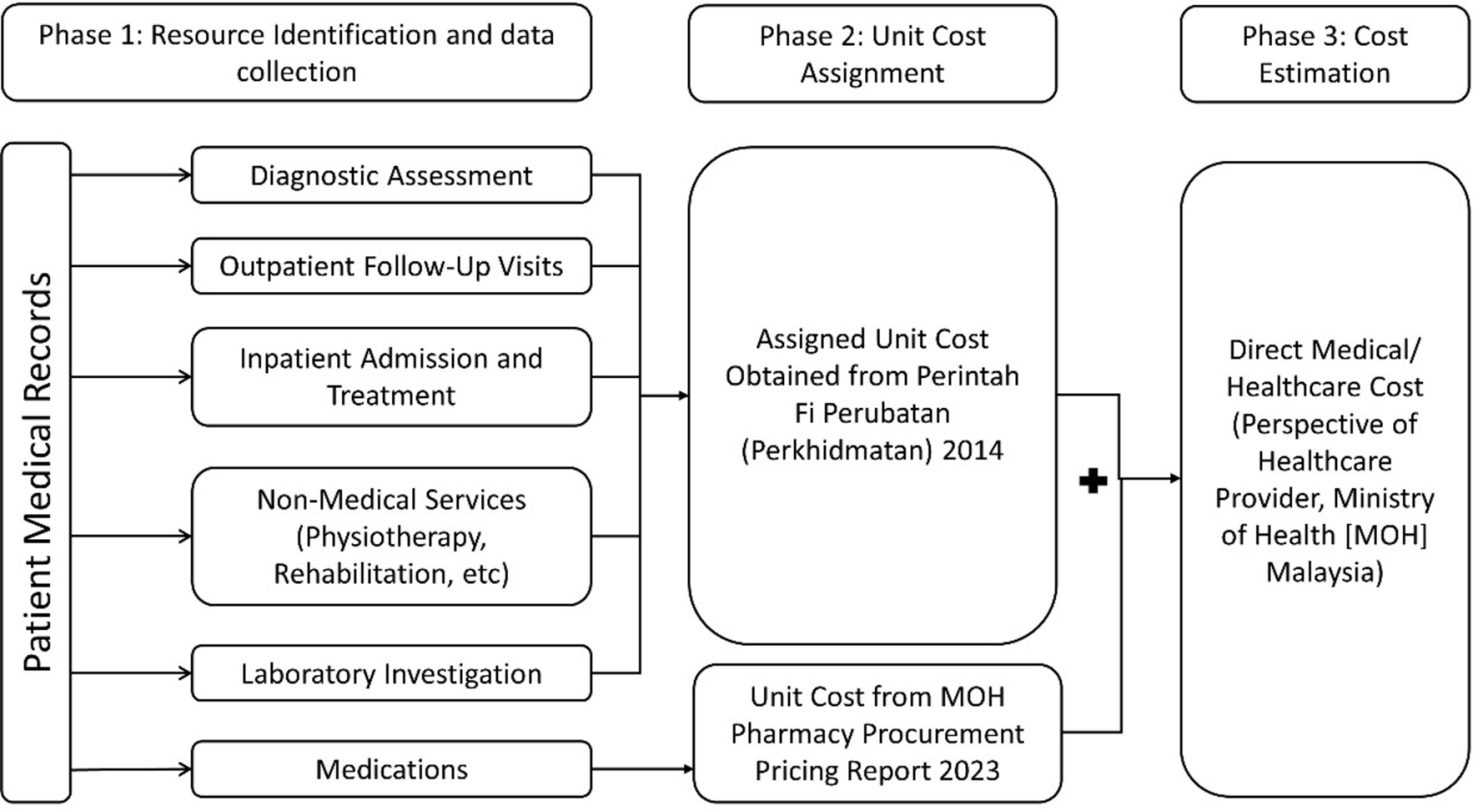
Structure of bottom-up micro-costing approach in estimating direct healthcare cost of Alzheimer's disease in this study.

In this prevalence-based study, bottom-up micro costing approach was utilized to estimate the disease burden. Prevalence based approach estimates the economic burden of a condition in a given or specified period of time, usually 1 year^25^. Next, bottom-up micro-costing approach was chosen as it is more detailed and precise to collect individual level utilization data during cost calculation^26^. In bottom-up micro-costing approach, three essential steps were taken to generate cost estimate: resource identification, unit measurement, and the valuation of resources, as illustrated in Fig. 2. To generate annual cost estimates, frequency of healthcare resource use was multiplied by the unit cost assigned to obtain estimated healthcare costs among people with AD^27^.

Unit cost of each healthcare resource were obtained from the MOH’s full paying patient scheme provided by Finance Department for the MOH hospitals^28^. Medication costs were estimated from the average acquisition cost from the Procurement Pricing Report provided by the Pharmacy Department’s Procurement Unit. All cost were converted to 2023 values based on Malaysian Consumer Price Index and expressed in Malaysian Ringgit (RM) and US Dollar (USD) with the conversion rate of USD1 = RM4.614 (dated August 2023). Calculations were done through the usage of self-designed costing template in Microsoft Excel 2023 version.

### Ethical approval

This study was designed adhering to WMA declaration of Helsinki^29^ and ethically approved by the Medical Research Ethics Committee Malaysia (MREC) with serial no of NMRR-ID-21-02014-VCP(IIR) to collect retrospective data from the hospitals. As there is no direct involvement of patients in the study, informed consent was waived by MREC along with ethical approval.

### Statistical analysis

The data was analysed using IBMSPSS Statistics 27 and Microsoft Excel. Categorical variables were expressed in numbers and percentages, while continuous variables were reported as mean [standard deviation (SD)] or median [Interquartile range (IQR)]. Outcomes measured and cost estimates were presented as mean (SD) or median [IQR] accordingly. Cost estimates of each AD patient were presented in terms of the DHC of AD per patient per year (PPPY). Inferential statistic tests were conducted including parametric tests [t-test, the analysis of variance (ANOVA)] and non-parametric tests if required [Mann–Whitney U test and Kruskal–Wallis H test]. To identify the cost predictors for the annual healthcare cost of AD, a generalised linear model (GLM) with log link and gamma distribution was employed in multivariate analysis due to the positively skewed cost data. The exponential of coefficients [Exp (B)] with 95% confidence interval (CI) were presented for each variable. A confidence interval of 95% with a p-value of < 0.05 was set to report Exp (B).

## Results

### Socio-demographic characteristics of Alzheimer’s disease subjects

A total of 333 patients with an average age of 77.4 ± 6.75 years were included from the six tertiary public hospital study sites. Large proportion of study participants were female (67.0%), aged between 71–80 years old (45.0%), of Chinese ethnicity (84.1%), had moderate AD (43.8%), had 3–4 comorbidities (38.7%), and stayed with their children (34.5%). The mean Mini Mental State Examination (MMSE) score was 17.6 ± 5.8. A majority of the patients had hypertension (62.5%), dyslipidaemia (48.3%), and diabetes mellitus (36.9%) These characteristics are summarised in Table 1.

**Table 1:** Basic sociodemographic characteristics of the study population.

Characteristics	N = 333
Gender (n, %)	
Male	110 (33.0)
Female	223 (67.0)
Study site (n, %)	
* East Malaysia*	123 (36.9)
Pusat Jantung Sarawak	123 (36.9)
* Penisular Malaysia*	210 (63.1)
Hospital Pulau Pinang	150 (44.4)
Hospital Seberang Jaya	20 (5.9)
Hospital Taiping	30 (8.9)
Hospital Tengku Ampuan Rahimah	4 (1.2)
Hospital Kuala Lumpur	6 (1.8)
Age in years (mean, SD)	77.41 (6.75)
Age range (n, %)	
65–70	61 (18.3)
71–80	150 (45.0)
81 and above	122 (36.6)
Ethnicity (n, %)	
Malay	26 (7.8)
Chinese	280 (84.1)
Indian	14 (4.2)
Others	13 (3.9)
Living condition (n, %)	
Spouse	92 (27.6)
Child	115 (34.5)
Sibling	9 (2.7)
SpA	48 (14.4)
Alone	12 (3.6)
Others	3 (0.9)
Spouse and child	48 (14.4)
Child and SpA	6 (1.8)
Disease severity (n, %)	
Mild	133 (39.9)
Moderate	146 (43.8)
Severe	54 (16.2)
MMSE score^a^ (mean, SD)	17.61 (5.98)
MMSE range (n, %)	
21–26	87 (32.8)
11–20	145 (54.7)
≤ 10	33 (12.5)
Comorbidity (mean, SD)	2.89 (1.72)
Comorbidity range (n, %)	
0	26 (7.8)
1–2	117 (35.1)
3–4	129 (38.7)
> 4	61 (18.3)
Common comorbidities (n, %)	
Hypertension	208 (62.5)
Type 2 diabetes mellitus	123 (36.9)
Dyslipidemia	161 (48.3)
Mental/brain disorder	76 (22.8)
Bone disease	83 (24.9)
Heart disease	66 (19.8)
Others	3–42 (0.9–12.6)

*SD* Standard deviation, *n* frequency, *%* percentage, *SpA* special accommodation, N denotes total patients; ^a^In MMSE score, the response rate was 79.58% where N = 265.

### Healthcare resource utilization and expenditure per patient per year

The mean (± SD) total DHC of AD PPPY was estimated RM 2641.30 (± 2133.35). Healthcare resource utilisation is summarised in Table 2*.* Laboratory tests (37.2%), clinic visits (31.48%), and medication (24.9%) make up most of the annual DHC. The mean DHC PPPY for mild, moderate, and severe stages of AD were RM2459.04 (± 1758.09), RM2642.27 (± 2213.72), and RM3087.61 (± 2667.90) respectively as shown in Table 3. While there was a trend towards increased annual DHC with increasing disease severity, these differences were not statistically significant (p = 0.19). The increasing costs are likely contributed by higher costs of clinic visits with increasing dementia severity, owing to more frequent clinic visits in patients with more severe stages of dementia. Conversely, a declining trend was detected in the mean cost of non-medical services from mild (RM109.25, ± 414.47), moderate (RM61.37, ± 295.41), to severe AD (RM21.48, ± 96.96).

**Table 2:** Healthcare resource utilization and cost distribution of the study population.

Variables	Total utilization, n	Frequency PPPY, mean (SD); median [IQR]	Cost PPPY(RM), mean (SD); median [IQR]	% of cost PPPY
Diagnostic assessment	793.00	2.38 (1.94)	95.02 (76.74)	3.60
		2 [1, 3.5]	80 [40, 140]	
Clinic visits	1532.00	4.60 (1.98)	831.59 (193.71)	31.48
Hospitalization (bed-day)	358.00	1.08 (4.38)	480 [360, 720]	
Non-medical services	568.00	1.71 (7.87)	74.02 (330)	2.80
Laboratory test	3241.00	9.73 (8.57)	982.18 (864.15)	37.19
		8 [4, 13]	874 [320.5, 1445.5]	
Medication	148,173.50	444.97 (324.81)	658.49 (1214.89)	24.93
		365 [275.13, 597.5]	127.75 [73, 657]	
Total direct healthcare cost, RM	–	–	2641.30 (2133.35)	
			2127.75 [1346.98, 3065.55]	

*PPPY* per patient per year, *SD* Standard deviation, *IQR* Interquartile range, *%* percentage, *n* frequency.

**Table 3:** Direct healthcare cost distribution among patient with AD in Malaysia stratified by disease severity.

Variables	Mild (n = 133)		Moderate (n = 146)		Severe (n = 54)	
	Cost PPPY (RM), Mean (SD); Median [IQR]	% of cost PPPY	Cost PPPY (RM), Mean (SD); Median [IQR]	% of cost PPPY	Cost PPPY (RM), Mean (SD); Median [IQR]	% of cost PPPY
Diagnostic assessment	101.05 (84.94)	4.10	102.47 (74.57)	3.87	60.00 (47.11)	1.94
	80 [120]		80 [120]		40 [40]	
Clinic visits and hospitalization	570.08 (287.87)	23.18	862.74 (1248.65)	32.65	1391.48 (1990.09)	45
	480 [360]		480 [360]		600 [750]	
Non-medical services	109.25 (414.47)	4.44	61.37 (295.41)	2.32	21.48 (96.96)	0.7
Laboratory test	882.23 (724.59)	35.877	1057.47 (898.36)	40.02	1024.83 (1058.12)	33.19
	756 [1110.50]		914 [1154.50]		894.50 [1161.75]	
Medication	796.44 (1540.81)	32.39	558.23 (956.43)	21.13	589.81 (865.96)	19.10
	113.89 [564.70]		148.19 [583.85]		236.39 [664.03]	
Total DHC	2459.04 (1758.09)	100	2642.27 (2213.72)	100	3087.61 (2667.90)	100
	2126.75 [1771.13]		2050.70 [1639.35]		2253.88 [1866.09]	

*PPPY* per patient per year, *DHC* Direct healthcare cost, *SD* Standard deviation, *IQR* Interquartile range, *%* percentage, *n* frequency.

### Annual DHC of Alzheimer’s Disease with Different Patient Characteristics

In terms of gender, difference in female (RM2720.25 ± RM2136.16) and male (RM2481.26 ± RM 2128.36) was not significant in either mean (p = 0.337) or median (p = 0.09) DHC PPPY. Followed by age group, the mean total DHC of AD PPPY for those aged 81 and above (RM 3119.24 ± RM2486.48) was found to be significantly higher (p = 0.008) than those aged 71 to 80 (RM 2344.90 ± RM 1842.86). Patients aged 65 to 70 also recorded lower mean DHC PPPY (RM2414.31 ± RM1882.74) than those aged 81 and above but slightly higher than those aged 71–80 as shown in Table 4. Besides, Indian patients (RM2470.49 ± RM2631.85) recorded a lower mean figure than Malay (RM2691.11 ± RM2054.63) and Chinese (RM2635.41 ± RM2151.11) while the highest figures (RM2852.53 ± RM1394.41) was recorded in other ethnicities despite no statistical difference detected.

**Table 4:** Annual Cost of Alzheimer’s Disease among AD patients with different characteristics.

Conditions	Frequency, (%)	Cost PPPY (RM), mean (SD)	Cost PPPY (RM), median [IQR]
Gender			
Male	110 (33)	2481.26 (2128.36)	1888.88 [1772.96]
Female	223 (67)	2720.25 (2136.16)	2218.55 [1735.70]
Age range			
65–70	61 (18.2)	2414.31 (1882.74)	1907.75 [1671.18]
71–80*	150 (45)	2344.90 (1842.86)	1991.84 [1421.53]
81 and above^R^	122 (36.6)	3119.24 (2486.48)	2404.58 [2235.19]
Ethnicity			
Malay	26 (7.8)	2691.11 (2054.63)	1960.61 [1492.84]
Chinese	280 (84.1)	2635.41 (2151.11)	2110.31 [1721.64]
Indian	14 (4.2)	2470.49 (2631.85)	1970.05 [2139.45]
Others	13 (39)	2852.53 (1394.41)	2950.08 [1295.10]
Living condition			
Spouse^R1^	92 (27.6)	2227.98 (1506.79)	1988.15 [1785.80]
Child^##2^	115 (34.5)	2596.75 (2163.36)	2127.75 [1707.50]
Sibling^##2,3^	9 (2.7)	2636.04 (2717.77)	2001 [1841.26]
SpA^##1^	48 (14.4)	3153.17 (2376.47)	2232.60 [2147.70]
Alone^R3^	12 (3.6)	3553.89 (2145.96)	2905.93 [1801.53]
Others	3 (0.9)	1866.45 (432.30)	1748.45 [-]
Spouse and Child^##2,3^	48 (14.4)	2657.56 (2522.10)	1910.24 [1791.49]
Child and SpA^R2,##1^	6 (1.8)	4178.09 (2892.24)	3337.50 [2727.68]
MMSE			
21–26	87 (32.8)	2894.41 (1962.17)	2414.35 [2305.20]
11–20	145 (54.7)	2716.24 (2273.39)	2151.48 [1649.03]
≤ 10	33 (12.5)	2722.68 (1948.59)	2416.70 [1612.53]
Comorbidity			
0	26 (7.8)	3235.22 (2759.69)	2513.33 [1474.57]
1–2	117 (35.1)	2400.11 (1961.47)	1986.55 [1688.84]
3–4	129 (38.7)	2731.43 (2186.91)	2249 [1931.10]
> 4	61 (18.3)	2660.19 (2021.08)	2126.75 [1625.37]
Comorbidity			
Hypertension	208 (62.5)	2644.51 (2162.26)	2091.65 [1712.56]
T2DM	123 (36.9)	2598.75 (2222.44)	1907.75 [1873.80]
Dyslipidaemia	161 (48.3)	2598.59 (2128.89)	2161.50 [1821.80]
Bone disease	83 (24.9)	2632.75 (1814.83)	2151.15 [1694.60]
Mental/brain disorder	76 (22.8)	2752.26 (1889.52)	2203.48 [2129.75]
Heart disease	66 (16.8)	2456.03 (1524.61)	2003.03 [1641.83]
Centres, n (%)*			
PJS*^1,#1,2^	123 (36.9)	2913.61 (2171.82)	2345.50 [1805.90]
HPP^##1,2^	150 (44.4)	2790.48 (2229.38)	2304.38 [1605.21]
HSJ^R2^	20 (5.9)	1638.81 (1580.04)	1447.68 [1167.64]
HTPG^R1^	30 (8.9)	1564.09 (1082.91)	1324.6 [121257]
HTAR	4 (1.2)	2552.16 (3220.48)	1112.70 [5081.19]
HKL	6 (1.8)	2115.04 (1636.49)	1317.20 [2568.36]

*PPPY* Per patient per year, *SpA* Special accommodation, *PJS* Pusat Jantung Sarawak, *HPP* Hospital Pulau Pinang, *HSJ* Hospital Seberang Jaya, *HTPG* Hospital Taiping, *HTAR* Hospital Tengku Ampuan Rahimah, *HKL* Hospital Kuala Lumpur, *T2DM* Type 2 Diabetes Mellitus *p < 0.01 in ANOVA or post hoc Bonferroni test, **p < 0.05 in ANOVA or post hoc Bonferroni test, ^#^p < 0.01 in Kruskal–Wallis test or Mann–Whitney test (non-parametric pairwise comparison test), ^##^p < 0.05 in Kruskal–Wallis test or Mann–Whitney test (non-parametric pairwise comparison test), ^R^reference item in post hoc test from either parametric (ANOVA) or non-parametric (Kruskal–Wallis) test.

On the other hand, AD patients who were staying with children and special accommodation recorded a mean annual DHC of RM4178.09 (± RM2892.24). AD patients who stayed alone spent an average of RM3553.89 ± RM2145.96 per year in healthcare resources followed by those who stayed in special accommodation (RM3155.17 ± RM2376.47) which was insignificant (p = 0.309 and 0.613 respectively) comparing with those who stayed in both child and special accommodation. Nevertheless, living conditions were not a significant factor (p = 0.10) towards the mean annual DHC of AD among older people. If median cost was discussed, statistical significance (p = 0.023) was observed in multiple comparisons. When staying with spouse was set as reference, medial annual DHC of AD was significantly lower than those who stayed with both children and special accommodation (RM3337.50 ± RM2727.68, p = 0.012), alone (RM2905.93 ± RM1801.53, p = 0.008) and in special accommodation only (RM2232.60 ± RM2147.70, p = 0.034). Compared with those with both children and special accommodation, median annual DHC of AD was significantly higher than those who stayed with children (RM2127.75 ± RM1707.50, p = 0.027), siblings (RM2001 ± RM1841.26, p = 0.044), and with both spouse and child (RM1910.24 ± RM1791.49, p = 0.017). AD patients who stayed alone recorded a significantly higher median annual healthcare cost than those who stayed with both spouse and children (p = 0.013) and with siblings (p = 0.023).

MMSE score was not significantly influencing the mean (p = 0.82) or median (p = 0.354) annual DHC of AD in the study population. Looking into the mean figures, AD patients with MMSE score between 21–26 had higher mean annual DHC of AD (RM2894.41 ± RM1962.71) than those between 11 and 20 (RM2716.24 ± RM2273.39) and 10 or below (RM2722.68 ± RM1948.59). On the other side, median annual DHC of AD was similar in those in MMSE scores between 21 and 26 (RM2414.35 ± RM2305.20) and 10 or below (RM2416.70 ± RM1612.53) while those recorded MMSE score between 11 and 20 was relatively lower (RM2151.48 ± RM1649.03).

The mean annual DHC of patients without any comorbidity (RM3235.22 ± RM2759.69) was recorded as the highest compared to those with comorbidities. For patients with one or 2 comorbidities, RM2400.11 (± RM1961.47) was spent on direct healthcare resource utilization in public hospitals. Such figures increased to RM2731.43 (± RM2186.91) when AD patients have 3–4 comorbidities. When AD patient had recorded more than 4 comorbidities, their expenditure on direct healthcare resources recorded at RM2660.19 (± RM2021.08). Among listed comorbidities, hypertension, type II diabetes mellitus and dyslipidemia were the top three common comorbidities among patients with AD in the study population. Nevertheless, AD patients who had mental or brain disorder as one of the comorbidities was found to have the highest mean annual DHC (RM2752.26 ± RM1889.52). Followed by the second highest mean annual DHC for AD patient who had hypertension as one of the comorbidities was recorded RM2644.51 (± RM2162.26). For AD patients with either Type II diabetes mellitus or dyslipidemia, the mean annual DHC was similar, recorded RM2598.75 (± RM2222.44) and RM2598.59 (± RM2128.89) respectively. Additionally, patients with bone disease (RM2632.75 ± RM1814.83) and heart disease (RM2456.03 ± RM1524.61) also recorded a significant mean annual DHC of AD during the 1-year period. No statistical significance is detected in mean (p = 0.291) or median (p = 0.168) annual DHC of AD in terms of comorbidities.

Patients who received direct medical care in Pusat Jantung Sarawak (PJS) recorded the highest healthcare cost (RM 2913.61 ± RM2171.82) followed by those who received treatment and care in HPP (RM2790.48 ± RM2229.38). Although the sample size obtained was relatively small, patients from HTAR (RM2552.16 ± RM3220.48) and HKL (RM2115.04 ± RM1636.49) have recorded higher annual mean DHC than those in HSJ (RM1638.81 ± RM1580.04) and HTPG (RM1564.09 ± RM1082.91). When detecting statistical significance, mean DHC of AD PPPY was significantly higher in PJS than in HTPG (p = 0.026). In median terms, patients in HSJ (RM 1447.68 ± RM1167.64) and HTPG (RM 1112.70 ± RM5081.19) spent significantly lower than those in PJS (RM 2345.50 ± RM1805.90, p = 0.002 and < 0.001 respectively) and HPP (RM 2304.38 ± RM1605.21, p = 0.007 and 0.002 respectively).

### Factors influencing DHC of Alzheimer’s disease

Table 5 illustrates the outcome of generalised linear model with log link function and gamma distribution. Seven variables were input into the generalised linear model namely gender, age range, ethnicity, number of comorbidities, living conditions, study sites and disease severity. Most of these variables were non-significant predictor for the mean annual DHC of AD except for patients age range. Compared with patients with AD aged 81 and above, the mean annual DHC of AD among those aged 71–80 years old was significantly lower by almost a quarter (Exp (B) = 0.780, 95% CI 0.661–0.919, p = 0.003).

**Table 5:** Predictors of mean annual DHC of AD among patients with AD in Malaysia.

Variables	Exp (B)	95% confidence interval	p-value
Demographics			
Female	0.994	0.843–1.172	0.945
Male	R	–	–
Age 65–70	0.859	0.696–1.085	0.214
Age 71–80	0.780	0.661–0.919	0.003*
Age > 80	R	–	–
Malay	1.156	0.723–1.848	0.545
Chinese	0.934	0.644–1.354	0.719
Indian	0.899	0.538–1.501	0.684
Others	R	–	–
Disease severity			
Mild	0.940	0.758–1.166	0.572
Moderate	0.916	0.739–1.134	0.419
Severe	R	–	–
Number of comorbidities			
0	1.281	0.926–1.771	0.134
1–2	1.051	0.844–1.310	0.655
3–4	1.078	0.877–1.325	0.477
> 4	R	–	–
Living condition			
Spouse	0.749	0.429–1.307	0.309
Child	0.754	0.442–1.288	0.302
Siblings	0.850	0.432–1.673	0.638
Special accommodation	0.867	0.500–1.505	0.613
Others	0.546	0.217–1.374	0.199
Alone	1.05	0.555–1.983	0.882
Spouse and Child	0.739	0.419–1.302	0.295
Child and special accommodation	R	–	–
Study site			
Pusat Jantung Sarawak	1.536	0.830–2.842	0.172
Hospital Pulau Pinang	1.414	0.772–2.588	0.262
Hospital Seberang Jaya	0.937	0.481–1.829	0.850
Hospital Taiping	0.861	0.459–1.615	0.642
Hospital TAR	1.168	0.501–2.725	0.719
Hospital Kuala Lumpur	R	–	–

*Denotes statistical significance (p < 0.05), R denotes reference value, Exp (B) Exponential beta.

## Discussion

This is the first study to utilise bottom-up micro-costing approach in estimating the direct medical cost of AD in Malaysia from older population aged 65 and above. The findings in this study filled the knowledge gap of economic cost of AD in receiving direct healthcare from public healthcare providers particularly Ministry of Health Malaysia. Aligning with the main objective, we found that the mean DHC of AD estimated from the study population was RM 2641.30 (USD 572.45) PPPY. Compared with neighboring countries, direct healthcare expenditure of AD in Malaysia was lower than that in Thailand and Singapore^11,12^ but greater than in the Philippines.^13^ This could be explained by the variations between countries in clinical practice of care, healthcare policy and social norms. For example, the unit cost of healthcare resource is higher in Thailand and Singapore when compared to Malaysia. Due to the non-for-profit nature of the MOH facilities, the rate of each healthcare service and resource has not been much revised since 2014. With that, the medical expenditure of AD patients in Singapore was recorded higher despite having a similar number of outpatient visits compared with Malaysia^11^. Such cost disparity was greater in comparison with Thailand as their AD patients visited memory clinics more often than in Malaysia^12^.

A previous local study reported mean cost of mild, moderate and severe dementia cases per episode of hospitalisation care was RM 8182 (SD 4811), RM 10,300 (SD 7190) and RM14,034 (SD 11,419), respectively, without further information about the annual cost of treatment^19^. Top-down approach was applied in this study where questions have been raised about such methodology compared with the bottom-up micro-costing approach in terms of feasibility, accuracy and reliability^26^. Although it is easy to conduct and time-saving, the top-down approach assesses cost starting from a national budget, which might not reflect the actual healthcare resource usage by specific disease during the time frame^30^. Compared to studies done in other regions, annual DHC in the current study was far lower than reported in other AD population, probably due to differences in the study design, resource availability and unit cost of resources in different settings^14,31–34^. In Spain and Japan, a prospective study design was used to track the pattern of healthcare resource utilization in estimating cost of AD among the study population reported an expenditure of USD3534.25 to USD5630.64, respectively in 18 months, which are much higher than in Malaysia^14,34^. In European and developed countries, the use of branded medications and the higher wage rate of healthcare professionals could raise the unit cost of drugs and clinic visits, respectively^14,31,32,34^.

Among different stages of AD, an increasing trend could be observed from mild to severe stage in estimating mean DHC per patient per year, which is consistent with several studies^31,33,35,36^. As patient’s condition worsens, the ability to perform daily activities and behavioural control is diminished, which requires more medical attention in alleviating behavioural or psychological symptoms (BPSD), such as medications and physiotherapy^34,36–38^. Nevertheless, other country estimates were inconsistent while estimating the DHC of AD based on disease severity^12,14,34,38–40^. For instance, an opposite trend was observed which DHC decreases from mild to moderate-severe AD in another Italy study. As mentioned in their limitation, such findings should be interpreted with caution due to the small sample size^40^.

Different trends in patient resource use were also observed in each disease severity, which were consistent with findings from GERAS studies conducted in France, UK, and Germany^35,39,41^. In our findings, patients with severe AD recorded higher frequency of outpatient visits as they required more clinical attention and care due to functional difficulties or BPSD which is similar in a multinational study^31^. Lower amount of diagnostic assessments recorded among severe patient with AD were due to several factors such as passive attitude from patients and short consultation time in public healthcare facilities. In addition, severe patient with AD had their functional ability worsened, which may lead to increased inpatient admission and treatment due to several medical issues such as cerebrovascular atrophy, recent falls, delirium and aggressive behaviour or multiple comorbidities as well as reflective of the lack of social support for the patients^42,43^. Besides, no clear trend in medication usage was observed across disease severity in the study population.

However, expenditure on prescription was a major component in annual mean DHC of AD in neighbouring countries^11–13^. While estimating AD medication cost, mild patient with AD recorded highest medication cost followed by severe and moderate patients in Malaysia. This could be explained by difference in prescribing pattern between specialists and procurement price of anti-Alzheimer’s medicine in government hospitals. Based on the procurement report of logistic pharmacy under MOH, rivastigmine in patch formulation was expensive than other medications such as donepezil and memantine. Not only that, use of rivastigmine patch in mild patient with AD was observed greater than donepezil or memantine due to either patient or prescriber factors such as compatibility issues, adverse drug reaction reduction and stability of disease control.

In the Andersen’s behavioural model, age, gender, race, and education were categorised as predisposing factors influencing healthcare resource utilization^44^. Through statistical analysis, patient sociodemographic such as gender, race, number of comorbidities, and living conditions was not significant in predicting DHC of patient with AD. In our study population, women (67%) recorded a major proportion in the diagnosis of Alzheimer’s Disease Related Dementia (ADRD). Despite of high prevalence of ADRD in women, this predisposing factor was not significant in predicting DHC of AD in this study. Such finding is consistent with other COI studies^12,14,31,36,38^. Such scenario could possibly explained by the longer survival age of women than men^45^ and other biological mechanisms^46,47^. In addition, the average age of patients with AD in Malaysia is 77.4 years old, which is slightly lower than Japan (80.3 years), Thailand (80.1 years), England (78.5 years), France (79.4 years) but higher than China (74 years), Argentina (74.5 years)^12,14,33,35,39,48^. Although AD is not a part of normal ageing, there is increasing risk of having AD once an individual reaches 65 years old^47^.

However, patient age group was identified to be a significant factor affecting DHC of AD in the study population. Expenditure on Alzheimer’s care increased considerably (p < 0.01) when patients with AD reached 80 years old in the study. Country-specific cost studies were consistent with such finding in their sample pool via survey data set or population based cohort data^49–51^. This could be explained as increasing age could raise the prevalence of chronic medical comorbidities and cognitive frailty such as cerebrovascular atrophy as well as tendency to fall and fragility fracture^52–54^. This is evident in the study as a quarter of study population has bone disorders. As it affects the mobility and daily function of older people, the impact of such age-related diseases should be weighed considerably^55,56^. Nevertheless, a systematic review done in 2014 found that the impact of age towards dementia cost is inconsistent of which further studies would be needed to investigate this finding^9^.

Apart from this, 57% of the study population has at least 3 medical comorbidities that requires regular follow-up in healthcare institutions. Among them, hypertension (62.5%) was common as well as dyslipidaemia (48.3%) and diabetes mellitus (36.9%). This finding was found similar in a study population in Spanish primary care centres. Considering the high prevalence of chronic diseases among older population, it is not surprising that patient with AD have an average of 2–8 comorbidities which could further affects the cognitive and functional ability in daily life^57,58^. Multiple pathological and environmental connections of such acquired risk factors could bring negative influence that accelerates the progression of AD and further increase healthcare demand in AD population^59^.

Significance of comorbidity towards associated cost in AD was observed in other studies^38^. Findings from China found that a surge of 18% in cost of care was anticipated in each additional one comorbidity^60^. In an ALSOVA study in Sweden, they found significant link between comorbidity and total cost of AD as well as other key indicators of disease^32^. Nonetheless, no statistical significance was observed between medical comorbidity and DHC of AD in this study population. Such findings were also observed in other cross-sectional studies^40^. This could be explained by methodological differences where in this study, data collection of healthcare resource use was limited to AD only instead of considering that of other major comorbidities too. Furthermore, willingness to healthcare in patient with AD could partially explain this finding. Despite suffering from many comorbidities, patient with AD may not reach out to healthcare providers due to many factors such as communication barrier, reluctance to cooperation and heavy reliance to informal caregivers^58^.

### Strength

Strength of this study includes the provision of updated real-world evidence in estimating DHC of AD in local patient with AD. Compared to previous attempt to quantify the financial burden of dementia in Malaysia, bottom-up micro costing approach offers data accuracy and reliability by utilizing actual patient medical records in data collection. As it is from a public healthcare payers’ perspective, such cost estimates could aid in formulating healthcare policy and enhance the process of decision making. Future cost-effectiveness analysis could be conducted with this cost estimate to identify potentials of interventions in delaying progression of AD or preventing permanent institutionalization.

### Limitations

On the other hand, this study has several limitations which need to be considered. First, there was no data collected from southern part of Malaysia which might underscore data representativeness of AD population in Malaysia. Additionally, the majority of subjects in the current study were of Chinese ethnicity, even though Malay is the majority ethnicity in Malaysia. This could partly be explained by almost half of the study subjects were from Penang state, where 40.3% of the population is Chinese^61^. Besides, Chinese and Indians are more likely to undergo medical check-ups than Malays among the elderly population according to a 2021 study^62^. This could also partly explain the ethnicity disparity observed in this study. Hence, the generalizability of the findings should be interpreted with caution. Due to retrospective nature of data collection method, some demographic information such as education status, smoking status and household income level were not investigated due to the unavailability of data. Besides, MMSE was recorded in only 79.58% of the study population while retrieving past medical records retrospectively. The unavailability of such data may be due to a few reasons, such as rejection towards test among patients, literacy issues, uncooperativeness of patients, age-associated diseases (such as hearing impairment or vision impairment) and/or loss of data. With that, it was reported descriptively but not included in subsequent GLM analysis. Future studies could consider a prospective study design with follow-up period that involves all country regions to detect potential shifts of cost over time in a representative sample. With prospective data collection method, future studies could include more potential demographic and clinical factors to identify potential relationships with AD cost. Nevertheless, the estimation in the current study could be an underestimate of the true cost of AD, which excludes the resource utilisation from other non-study sites’ facilities and overhead cost. Moreover, the societal perspective could be applied in future economic evaluations which covers out-of-pocket expenses from patients that could be more informative for the burden of AD to society as a whole.

## Conclusion

Real-world evidence is important in assisting the process of decision making and formulating healthcare policies in geriatric care. From public healthcare providers’ perspective, the annual mean DHC of AD is recorded RM2641.30 (USD 572.45) per patient. Increasing cost is anticipated when patient with AD advances to severe stages and age over time. The current study’s cost estimates could be useful in future cost- effectiveness studies and budget impact analysis to evaluate potential interventions of AD. Future studies could consider evaluating the resource use of patients with AD using a prospective approach to identify more possible associations. Resource use and cost in private healthcare facilities could be incorporated to enable discussion across healthcare settings.

### Supplementary Information

Supplementary Information.

## Data Availability

The datasets generated during and/or analysed during the current study are available from the corresponding author on reasonable request.
