# Supplementary material for: Direct healthcare expenditure on Alzheimer’s disease from healthcare providers’ perspective in Malaysia: a micro-costing approach
Source: Sci Rep. 2024 Aug 14;14:18855. doi: 10.1038/s41598-024-69745-1 (PMC11324753; doi:10.1038/s41598-024-69745-1)
Supplement: Supplementary file 1 — Supplementary Information. [file 41598_2024_69745_MOESM1_ESM.pdf]

Manuscript Type: Original Research Article

Direct Healthcare Expenditure on Alzheimer's Disease from Healthcare Providers' Perspective in Malaysia: A Micro-Costing Approach

Siew Chin Ong*[1], Lyn Xuan Tay[1], Teck Fah Yee[2], Ewe Eow Teh[3], Alan Swee Hock Ch'ng[4], Rizah Mazzuin Razali[5], Wan Chieh Lim[6], Ungku Ahmad Ameen Bin Ungku Mohd. Zam[7], Thaigarajan Parumasivam[8]

Affiliations:

1. Discipline of Social and Administrative Pharmacy, Universiti Sains Malaysia, 11800 Pulau Pinang, Malaysia.
2. Pharmacy Department, Hospital Queen Elizabeth, Ministry of Health Malaysia, 88586, Kota Kinabalu, Sabah, Malaysia.
3. Department of Psychiatry & Mental Health, Hospital Pulau Pinang, Ministry of Health Malaysia, 10990 Pulau Pinang, Malaysia
4. Department of Medicine, Seberang Jaya Hospital, Ministry of Health Malaysia, 13700 Seberang Perai, Penang, Malaysia.
5. Geriatric Unit, Department of Medicine, Kuala Lumpur Hospital, Ministry of Health Malaysia, 50586, Kuala Lumpur, Malaysia.
6. Geriatrics Unit, Internal Medicine Department, Taiping General Hospital, Ministry of Health Malaysia, 34000, Taiping, Perak, Malaysia.
7. Geriatrics Unit, Medical Department, Hospital Tengku Ampuan Rahimah Klang, Ministry of Health Malaysia, 41200 Klang, Selangor, Malaysia.
8. Discipline of Pharmaceutical Technology, Universiti Sains Malaysia, 11800 Pulau Pinang, Malaysia.

Corresponding author: Siew Chin Ong, Dr. (ORCID: 0000-0002-9750-9588)

Email: siewchinong@usm.my

Tel: +6012-4770883

Supplementary Materials

S1. List of Assessment tests done during outpatient follow-up visits

MMSE: Mini Mental State Examination

MBI: Modified Barthel Index

GDS: Global Deterioration Scale

HADS: Hospital Anxiety and Depression Scale

CSDD: Cornell scale of Depression in Dementia

NPI: Neuropsychiatry Index

CDT: Clock Drawing Test

BDI: BrainTrip Dementia Index (Test early signs of dementia/early stages)

VCAT: Visual Cognitive Assessment Test

BPRS14/24: Brief Psychiatric Rating Scale-14 pr 24 item

S2. List of comorbidities

Category	Items
Hypertension	Labelled as HPT, HTN, sometimes typo as HOT
DM	Type or 2 DM, Impaired glucose tolerance also categorised into this
Lipid disorder	Dyslipidaemia (DLP), Hypercholesterolemia, or any other mentioned in CPG
Kidney Disorder	Acute Kidney Injury (AKI/AKD), Chronic Kidney Disease (CKD), Nephrolithiasis, Renal tubular acidosis (RTA), partial bladder rejection,
Bone Disorder	Osteopenia, Osteoporosis (OP), Rheumatoid Arthritis (RA), Degenerative Spine Disease, Gout, Bone Fracture, Cervical Myelopathy, Fall history
Brain Disorder/ Neurological disorder	Cerebral Vascular Atropy (CVA)/Stroke, Depression, Anxiety, Insomnia, Multifocal Infarct, Lacunar Stroke, cerebellar infarcts, brain stem stroke, subdural hematoma (SDH), parkinsonism, seizure, Trigeminal Neuralgia,
Eye Disorder	Unilateral/ Bilateral Cataract, Glaucoma, Blindness, diabetic retinopathy,
Heart Disease	Ischemia Heart Disease (IHD), Atrial Fibrillation (AF), Sick Sinus Syndrome, Congestive Heart Failure(CCF), Heart failure (HF), Coronary Artery Disease (CAD)
Lung Disease	Asthma, COPD, Bronchitis, Bronchial Asthma(BA), Pneumonia, Lung fibrosis, Pulmonary Tuberculosis (PTB), COVID-19, parapneumonic effusion
BPH/UI	Begnin Prostate Hyperplasia/ Urinary Incontinence
Blood Autoimmune Disorder	Anaemia, thalassemia, Deep Vein Thrombosis (DVT), Alcoholic ketoacidosis (AKA)
Tumour/ Cancer/ Viral Infection	Breast cancer, Prostate Cancer, Colorectal cancer, Lung tumour/ cancer, Hepatitis B/C, Syphillis, Nasopharyngeal Cancer (NPC)
Hearing Impairment	

Endocrine Disorder	Hyperthyroidism, Hypothyroidism, Throitoxicosis
Liver Disorder	Liver Cirrhosis, Jaundice
Surgical History	Total Knee replacement (TKR), Bilateral Eye Pseudophatic (BE Pseudo), Partial anterior circulation infarct (PACI), Thyroidectomy, cholecystectomy, hernioplasty
Others	Vitamin deficiency, Sjorgen syndrome, Eczema, Obstructive sleep apnoea (OSA), gastritis, GI Lymphoma, Uroprolapse, Skin Conditions.

S3. List Of Laboratory Investigations

Albumin (Blood/Urine)

Alpha feto protein (AFP)

Arterial blood gas (ABG)

Bilirubin

Blood Urea & Serum Electrolytes (BUSE)

Calcium Level

Cancer marker 125

Cancer marker 19.9

Carcinoembryonic Antigen (CEA)

Cardiac Enzymes

Cerebrospinal Fluid Analysis (CFA)

Computed Tomography (CT) Scan

Cortisol

C-Reactive Protein (CRP)

Creatine kinase-muscle bound (CK-MB)

Creatinine

D-Dimer

Doppler Echocardiogram

Dual X-Ray Absorptiometry (DEXA) Scan

Echocardiography (ECHO) transthoracic

Electrocardiogram (ECG)

Electroencephalogram (EEG)

Erythrocyte Sedimentation Rate (ESR)

Fasting Blood Glucose (FBG) / Fasting Blood Sugar (FBS)

Fluorodeoxyglucose Positron Emission Tomography (FDG PET) scan

Full Blood Count (FBC)

Full Blood Picture (FBP)

Full Lipid Profile (FLP)

Full Liver Function Test (LFT)

Haemoglobin A1C (HbA1C)

Hepatitis B surface Antigen

Hepatitis C Virus Antibody

HIV antigen test

Holter

International Normalised Ratio (INR)

Iron Serum (total)

L. Dehydrogenase test

Magnesium & Phosphate

Magnetic Resonance Imaging (MRI) Scan

Other Cytology (Bacteria CNS)

Partial Thromboplastin Time (PTT)

Potassium

Prostate-Specific Antigen (PSA) test

Prothrombin Time (PT)

Random Blood Glucose (RBG) / Random Blood Sugar (RBS)

Renal Profile(RP)

Serum Folate

Serum Vitamin B12

Stool Full Examination Microscopic Examination (Stool FEME)

Thyroid Function Test (TFT)

Ultrasound Scan (Abdomen/ Kidney, bladder, and residual urine volume estimation)

Uric Acid

Urine Cytology (Urine Culture and Sensitivity [UCNS])

Urine Full Examination Microscopy Examination (UFEME)

Urine Protein

Urine Protein Creatinine Index

Venereal disease research laboratory test (VDRL)

Venous blood gas (VBG)

X-Ray scan

S4. List of Medications included

Medication Class	Name of medication with dose and formulation
Alzheimer's disease prescription medicines	Rivastigmine 3 mg Capsule<br>Rivastigmine 4.6mg/24hr Transdermal<br>Rivastigmine 9.5 mg/24hr Transdermal<br>Memantine HCI 10 mg Tablet<br>Donepezil HCl 10 mg Tablet<br>Donepezil HCl 5 mg Tablet
Anti-Psychotics	Aripiprazole 10mg Tablet<br>Aripiprazole 400mg Prolonged-Release<br>Risperidone 1 mg Tablet<br>Quetiapine Fumarate 100mg IR Tablet<br>Quetiapine Fumarate 200mg IR Tablet<br>Olanzapine 10 mg DISINTEGRATING<br>Olanzapine 10 mg Tablet<br>Olanzapine 5mg DISINTEGRATING<br>Olanzapine 5 mg Tablet<br>Haloperidol 5mg Tablet<br>Haloperidol 5 mg/ml Injection<br>Haloperidol 1.5 mg Tablet
Antidepressants	Clomipramine HCI 25mg Tablet<br>Fluoxetine HCl 20 mg Capsule/Tablet<br>Sertraline HCI 50 mg Tablet<br>Escitalopram 10 mg Tablet<br>Fluvoxamine 100mg Tablet<br>Fluvoxamine 50mg Tablet<br>Mirtazapine 30 mg Orodispersible Tablet<br>Mirtazapine 15 mg Orodispersible Tablet
Others	Clonazepam 2 mg Tablet<br>Clonazepam 0.5 mg Tablet<br>Zolpidem Tartrate 10 mg Tablet<br>Alprazolam 0.5 mg Tablet

S5. Diagnostic and statistical manual of mental disorders: DSM-5 (Page 602-605, 611-614)

293.0 (F05) Delirium due to multiple etiologies
Specify if: Acute, Persistent
Specify if: Hyperactive, Hypoactive, Mixed level of activity

780.09 (R41.0) Other Specified Delirium (602)

780.09 (R41.0) Unspecified Delirium (602)

Major and Mild Neurocognitive Disorders (602)

Specify whether due to: Alzheimer's disease, Frontotemporal lobar degeneration, Lewy body disease, Vascular disease, Traumatic brain injury, Substance/medication use, HIV infection, Prion disease, Parkinson's disease, Huntington's disease, Another medical condition, Multiple etiologies, Unspecified

[a]*Specify* Without behavioral disturbance, With behavioral disturbance. *For possible major neurocognitive disorder and for mild neurocognitive disorder, behavioral disturbance cannot be coded but should still be indicated in writing.*

[b]*Specify* current severity: Mild, Moderate, Severe. *This specifier applies only to major neurocognitive disorders (including probable and possible).*

Note: As indicated for each subtype, an additional medical code is needed for probable major neurocognitive disorder or major neurocognitive disorder. An additional medical code should *not* be used for possible major neurocognitive disorder or mild neurocognitive disorder.

Major or Mild Neurocognitive Disorder Due to Alzheimer's Disease (611)

___.__ (___.__) Probable Major Neurocognitive Disorder Due to Alzheimer's Disease[b]
Note: Code first **331.0 (G30.9)** Alzheimer's disease.

294.11 (F02.81) With behavioral disturbance

294.10 (F02.80) Without behavioral disturbance

331.9 (G31.9) Possible Major Neurocognitive Disorder Due to Alzheimer's Disease[a, b]

331.83 (G31.84) Mild Neurocognitive Disorder Due to Alzheimer's Disease[a]

Major or Mild Frontotemporal Neurocognitive Disorder (614)

___.__ (___.__) Probable Major Neurocognitive Disorder Due to Frontotemporal Lobar Degeneration[b]
Note: Code first **331.19 (G31.09)** frontotemporal disease.

294.11 (F02.81) With behavioral disturbance

294.10 (F02.80) Without behavioral disturbance

331.9 (G31.9) Possible Major Neurocognitive Disorder Due to Frontotemporal Lobar Degeneration[a, b]

331.83 (G31.84) Mild Neurocognitive Disorder Due to Frontotemporal Lobar Degeneration[a]

Major or Mild Neurocognitive Disorder With Lewy Bodies (618)

___.__ (___.__) Probable Major Neurocognitive Disorder With Lewy Bodies[b]
Note: Code first **331.82 (G31.83)** Lewy body disease.

294.11 (F02.81) With behavioral disturbance

294.10 (F02.80) Without behavioral disturbance

Other Specified Delirium

780.09 (R41.0)

This category applies to presentations in which symptoms characteristic of delirium that cause clinically significant distress or impairment in social, occupational, or other important areas of functioning predominate but do not meet the full criteria for delirium or any of the disorders in the neurocognitive disorders diagnostic class. The other specified delirium category is used in situations in which the clinician chooses to communicate the specific reason that the presentation does not meet the criteria for delirium or any specific neurocognitive disorder. This is done by recording "other specified delirium" followed by the specific reason (e.g., "attenuated delirium syndrome").

An example of a presentation that can be specified using the "other specified" designation is the following:

Attenuated delirium syndrome: This syndrome applies in cases of delirium in which the severity of cognitive impairment falls short of that required for the diagnosis, or in which some, but not all, diagnostic criteria for delirium are met.

Unspecified Delirium

780.09 (R41.0)

This category applies to presentations in which symptoms characteristic of delirium that cause clinically significant distress or impairment in social, occupational, or other important areas of functioning predominate but do not meet the full criteria for delirium or any of the disorders in the neurocognitive disorders diagnostic class. The unspecified delirium category is used in situations in which the clinician chooses *not* to specify the reason that the criteria are not met for delirium, and includes presentations for which there is insufficient information to make a more specific diagnosis (e.g., in emergency room settings).

Major and Mild Neurocognitive Disorders

Major Neurocognitive Disorder

Diagnostic Criteria

A. Evidence of significant cognitive decline from a previous level of performance in one or more cognitive domains (complex attention, executive function, learning and memory, language, perceptual-motor, or social cognition) based on:
   1. Concern of the individual, a knowledgeable informant, or the clinician that there has been a significant decline in cognitive function; and
   2. A substantial impairment in cognitive performance, preferably documented by standardized neuropsychological testing or, in its absence, another quantified clinical assessment.

B. The cognitive deficits interfere with independence in everyday activities (i.e., at a minimum, requiring assistance with complex instrumental activities of daily living such as paying bills or managing medications).

C. The cognitive deficits do not occur exclusively in the context of a delirium.

D. The cognitive deficits are not better explained by another mental disorder (e.g., major depressive disorder, schizophrenia).

Specify whether due to:

Alzheimer's disease (pp. 611–614)
Frontotemporal lobar degeneration (pp. 614–618)
Lewy body disease (pp. 618–621)
Vascular disease (pp. 621–624)
Traumatic brain injury (pp. 624–627)
Substance/medication use (pp. 627–632)
HIV infection (pp. 632–634)
Prion disease (pp. 634–636)
Parkinson's disease (pp. 636–638)
Huntington's disease (pp. 638–641)
Another medical condition (pp. 641–642)
Multiple etiologies (pp. 642–643)
Unspecified (p. 643)

Coding note: Code based on medical or substance etiology. In some cases, there is need for an additional code for the etiological medical condition, which must immediately precede the diagnostic code for major neurocognitive disorder, as follows:

Etiological subtype	Associated etiological medical code for major neurocognitive disorder[a]	Major neurocognitive disorder code[b]	Mild neurocognitive disorder code[c]
Alzheimer's disease	Probable: 331.0 (G30.9) Possible: no additional medical code	Probable: 294.1x (F02.8x) Possible: 331.9 (G31.9)[c]	331.83 (G31.84) (Do not use additional code for Alzheimer's disease.)
Frontotemporal lobar degeneration	Probable: 331.19 (G31.09) Possible: no additional medical code	Probable: 294.1x (F02.8x) Possible: 331.9 (G31.9)[c]	331.83 (G31.84) (Do not use additional code for frontotemporal disease.)
Lewy body disease	Probable: 331.82 (G31.83) Possible: no additional medical code	Probable: 294.1x (F02.8x) Possible: 331.9 (G31.9)[c]	331.83 (G31.84) (Do not use additional code for Lewy body disease.)
Vascular disease	No additional medical code	Probable: 290.40 (F01.5x) Possible: 331.9 (G31.9)[c]	331.83 (G31.84) (Do not use additional code for the vascular disease.)
Traumatic brain injury	907.0 (S06.2X9S)	294.1x (F02.8x)	331.83 (G31.84) (Do not use additional code for the traumatic brain injury.)
Substance/medication-induced	No additional medical code	Code based on the type of substance causing the major neurocognitive disorder[c, d]	Code based on the type of substance causing the mild neurocognitive disorder[d]

Etiological subtype	Associated etiological medical code for major neurocognitive disorder[a]	Major neurocognitive disorder code[b]	Mild neurocognitive disorder code[c]
HIV infection	042 (B20)	294.1x (F02.8x)	331.83 (G31.84) (Do not use additional code for HIV infection.)
Prion disease	046.79 (A81.9)	294.1x (F02.8x)	331.83 (G31.84) (Do not use additional code for prion disease.)
Parkinson's disease	Probable: 332.0 (G20) Possible: No additional medical code	Probable: 294.1x (F02.8x) Possible: 331.9 (G31.9)[c]	331.83 (G31.84) (Do not use additional code for Parkinson's disease.)
Huntington's disease	333.4 (G10)	294.1x (F02.8x)	331.83 (G31.84) (Do not use additional code for Huntington's disease.)
Due to another medical condition	Code the other medical condition first (e.g., 340 [G35] multiple sclerosis)	294.1x (F02.8x)	331.83 (G31.84) (Do not use additional codes for the presumed etiological medical conditions.)
Due to multiple etiologies	Code all of the etiological medical conditions first (with the exception of vascular disease)	294.1x (F02.8x) (Plus the code for the relevant substance/medication-induced major neurocognitive disorders if substances or medications play a role in the etiology.)	331.83 (G31.84) (Plus the code for the relevant substance/medication-induced mild neurocognitive disorders if substances or medications play a role in the etiology. Do not use additional codes for the presumed etiological medical conditions.)
Unspecified neurocognitive disorder	No additional medical code	799.59 (R41.9)	799.59 (R41.9)

[a]Code first, before code for major neurocognitive disorder.
[b]Code fifth character based on symptom specifier: .x0 without behavioral disturbance; .x1 with behavioral disturbance (e.g., psychotic symptoms, mood disturbance, agitation, apathy, or other behavioral symptoms).
[c]**Note:** Behavioral disturbance specifier cannot be coded but should still be indicated in writing.
[d]See "Substance/Medication-Induced Major or Mild Neurocognitive Disorder."

Specify:

Without behavioral disturbance: If the cognitive disturbance is not accompanied by any clinically significant behavioral disturbance.

With behavioral disturbance *(specify disturbance):* If the cognitive disturbance is accompanied by a clinically significant behavioral disturbance (e.g., psychotic symptoms, mood disturbance, agitation, apathy, or other behavioral symptoms).

Specify current severity:

Mild: Difficulties with instrumental activities of daily living (e.g., housework, managing money).

Moderate: Difficulties with basic activities of daily living (e.g., feeding, dressing).

Severe: Fully dependent.

Mild Neurocognitive Disorder

Diagnostic Criteria

A. Evidence of modest cognitive decline from a previous level of performance in one or more cognitive domains (complex attention, executive function, learning and memory, language, perceptual-motor, or social cognition) based on:
   1. Concern of the individual, a knowledgeable informant, or the clinician that there has been a mild decline in cognitive function; and
   2. A modest impairment in cognitive performance, preferably documented by standardized neuropsychological testing or, in its absence, another quantified clinical assessment.

B. The cognitive deficits do not interfere with capacity for independence in everyday activities (i.e., complex instrumental activities of daily living such as paying bills or managing medications are preserved, but greater effort, compensatory strategies, or accommodation may be required).

C. The cognitive deficits do not occur exclusively in the context of a delirium.

D. The cognitive deficits are not better explained by another mental disorder (e.g., major depressive disorder, schizophrenia).

Specify whether due to:

Alzheimer's disease (pp. 611–614)
Frontotemporal lobar degeneration (pp. 614–618)
Lewy body disease (pp. 618–621)
Vascular disease (pp. 621–624)
Traumatic brain injury (pp. 624–627)
Substance/medication use (pp. 627–632)
HIV infection (pp. 632–634)
Prion disease (pp. 634–636)
Parkinson's disease (pp. 636–638)
Huntington's disease (pp. 638–641)
Another medical condition (pp. 641–642)
Multiple etiologies (pp. 642–643)
Unspecified (p. 643)

Coding note: For mild neurocognitive disorder due to any of the medical etiologies listed above, code **331.83 (G31.84).** Do *not* use additional codes for the presumed etiological medical conditions. For substance/medication-induced mild neurocognitive disorder, code based on type of substance; see "Substance/Medication-Induced Major or Mild Neurocognitive Disorder." For unspecified mild neurocognitive disorder, code **799.59 (R41.9).**

Major or Mild Neurocognitive Disorder Due to Alzheimer's Disease

Diagnostic Criteria

A. The criteria are met for major or mild neurocognitive disorder.

B. There is insidious onset and gradual progression of impairment in one or more cognitive domains (for major neurocognitive disorder, at least two domains must be impaired).

C. Criteria are met for either probable or possible Alzheimer's disease as follows:

For major neurocognitive disorder:

Probable Alzheimer's disease is diagnosed if either of the following is present; otherwise, **possible Alzheimer's disease** should be diagnosed.

1. Evidence of a causative Alzheimer's disease genetic mutation from family history or genetic testing.
2. All three of the following are present:
   a. Clear evidence of decline in memory and learning and at least one other cognitive domain (based on detailed history or serial neuropsychological testing).
   b. Steadily progressive, gradual decline in cognition, without extended plateaus.
   c. No evidence of mixed etiology (i.e., absence of other neurodegenerative or cerebrovascular disease, or another neurological, mental, or systemic disease or condition likely contributing to cognitive decline).

For mild neurocognitive disorder:

Probable Alzheimer's disease is diagnosed if there is evidence of a causative Alzheimer's disease genetic mutation from either genetic testing or family history.

Possible Alzheimer's disease is diagnosed if there is no evidence of a causative Alzheimer's disease genetic mutation from either genetic testing or family history, and all three of the following are present:

1. Clear evidence of decline in memory and learning.
2. Steadily progressive, gradual decline in cognition, without extended plateaus.
3. No evidence of mixed etiology (i.e., absence of other neurodegenerative or cerebrovascular disease, or another neurological or systemic disease or condition likely contributing to cognitive decline).

D. The disturbance is not better explained by cerebrovascular disease, another neurodegenerative disease, the effects of a substance, or another mental, neurological, or systemic disorder.

Coding note: For probable major neurocognitive disorder due to Alzheimer's disease, with behavioral disturbance, code first **331.0 (G30.9)** Alzheimer's disease, followed by **294.11 (F02.81)** major neurocognitive disorder due to Alzheimer's disease. For probable neurocognitive disorder due to Alzheimer's disease, without behavioral disturbance, code first **331.0 (G30.9)** Alzheimer's disease, followed by **294.10 (F02.80)** major neurocognitive disorder due to Alzheimer's disease, without behavioral disturbance.

For possible major neurocognitive disorder due to Alzheimer's disease, code **331.9 (G31.9)** possible major neurocognitive disorder due to Alzheimer's disease. (**Note:** Do *not* use the additional code for Alzheimer's disease. Behavioral disturbance cannot be coded but should still be indicated in writing.)

For mild neurocognitive disorder due to Alzheimer's disease, code **331.83 (G31.84).** (**Note:** Do *not* use the additional code for Alzheimer's disease. Behavioral disturbance cannot be coded but should still be indicated in writing.)

Diagnostic Features

Beyond the neurocognitive disorder (NCD) syndrome (Criterion A), the core features of major or mild NCD due to Alzheimer's disease include an insidious onset and gradual progression of cognitive and behavioral symptoms (Criterion B). The typical presentation is amnestic (i.e., with impairment in memory and learning). Unusual nonamnestic presentations, particularly visuospatial and logopenic aphasic variants, also exist. At the mild NCD phase, Alzheimer's disease manifests typically with impairment in memory and learning, sometimes accompanied by deficits in executive function. At the major NCD phase, visuoconstructional/perceptual-motor ability and language will also be impaired, particularly when the NCD is moderate to severe. Social cognition tends to be preserved until late in the course of the disease.

A level of diagnostic certainty must be specified denoting Alzheimer's disease as the "probable" or "possible" etiology (Criterion C). *Probable Alzheimer's disease* is diagnosed in both major and mild NCD if there is evidence of a causative Alzheimer's disease gene, either from genetic testing or from an autosomal dominant family history coupled with autopsy confirmation or a genetic test in an affected family member. For major NCD, a typical clinical picture, without extended plateaus or evidence of mixed etiology, can also be diagnosed as due to probable Alzl[illegible]r's disease. For mild NCD, given the lesser degree of certainty that the deficits will progress, these features are only sufficient for a *possible* Alzheimer's etiology. If the etiology appears mixed, mild NCD due to multiple etiologies should be diagnosed. In any case, for both mild and major NCD due to Alzheimer's disease, the clinical features must not suggest another primary etiology for the NCD (Criterion D).

Associated Features Supporting Diagnosis

In specialty clinical settings, approximately 80% of individuals with major NCD due to Alzheimer's disease have behavioral and psychological manifestations; these features are also frequent at the mild NCD stage of impairment. These symptoms are as or more distressing than cognitive manifestations and are frequently the reason that health care is sought. At the mild NCD stage or the mildest level of major NCD, depression and/or apathy are often seen. With moderately severe major NCD, psychotic features, irritability, agitation, combativeness, and wandering are common. Late in the illness, gait disturbance, dysphagia, incontinence, myoclonus, and seizures are observed.

Prevalence

The prevalence of overall dementia (major NCD) rises steeply with age. In high-income countries, it ranges from 5% to 10% in the seventh decade to at least 25% thereafter. U.S. census data estimates suggest that approximately 7% of individuals diagnosed with Alzheimer's disease are between ages 65 and 74 years, 53% are between ages 75 and 84 years, and 40% are 85 years and older. The percentage of dementias attributable to Alzheimer's disease ranges from about 60% to over 90%, depending on the setting and diagnostic criteria. Mild NCD due to Alzheimer's disease is likely to represent a substantial fraction of mild cognitive impairment (MCI) as well.

Development and Course

Major or mild NCD due to Alzheimer's disease progresses gradually, sometimes with brief plateaus, through severe dementia to death. The mean duration of survival after di-

agnosis is approximately 10 years, reflecting the advanced age of the majority of individuals rather than the course of the disease; some individuals can live with the disease for as long as 20 years. Late-stage individuals are eventually mute and bedbound. Death most commonly results from aspiration in those who survive through the full course. In mild NCD due to Alzheimer's disease, impairments increase over time, and functional status gradually declines until symptoms reach the threshold for the diagnosis of major NCD.

The onset of symptoms is usually in the eighth and ninth decades; early-onset forms seen in the fifth and sixth decades are often related to known causative mutations. Symptoms and pathology do not differ markedly at different onset ages. However, younger individuals are more likely to survive the full course of the disease, while older individuals are more likely to have numerous medical comorbidities that affect the course and management of the illness. Diagnostic complexity is higher in older adults because of the increased likelihood of comorbid medical illness and mixed pathology.

Risk and Prognostic Factors

Environmental. Traumatic brain injury increases risk for major or mild NCD due to Alzheimer's disease.

Genetic and physiological. Age is the strongest risk factor for Alzheimer's disease. The genetic susceptibility polymorphism apolipoprotein E4 increases risk and decreases age at onset, particularly in homozygous individuals. There are also extremely rare causative Alzheimer's disease genes. Individuals with Down's syndrome (trisomy 21) develop Alzheimer's disease if they survive to midlife. Multiple vascular risk factors influence risk for Alzheimer's disease and may act by increasing cerebrovascular pathology or also through direct effects on Alzheimer pathology.

Culture-Related Diagnostic Issues

Detection of an NCD may be more difficult in cultural and socioeconomic settings where memory loss is considered normal in old age, where older adults face fewer cognitive demands in everyday life, or where very low educational levels pose greater challenges to objective cognitive assessment.

Diagnostic Markers

Cortical atrophy, amyloid-predominant neuritic plaques, and tau-predominant neurofibrillary tangles are hallmarks of the pathological diagnosis of Alzheimer's disease and may be confirmed via postmortem histopathological examination. For early-onset cases with autosomal dominant inheritance, a mutation in one of the known causative Alzheimer's disease genes—amyloid precursor protein (APP), presenilin 1 (PSEN1), or presenilin 2 (PSEN2)—may be involved, and genetic testing for such mutations is commercially available, at least for PSEN1. Apolipoprotein E4 cannot serve as a diagnostic marker because it is only a risk factor and neither necessary nor sufficient for disease occurrence.

Since amyloid beta-42 deposition in the brain occurs early in the pathophysiological cascade, amyloid-based diagnostic tests such as amyloid imaging on brain positron emission tomography (PET) scans and reduced levels of amyloid beta-42 in the cerebrospinal fluid (CSF) may have diagnostic value. Signs of neuronal injury, such as hippocampal and temporoparietal cortical atrophy on a magnetic resonance image scan, temporoparietal hypometabolism on a fluorodeoxyglucose PET scan, and evidence for elevated total tau and phospho-tau levels in CSF, provide evidence of neuronal damage but are less specific for Alzheimer's disease. At present, these biomarkers are not fully validated, and many are available only in tertiary care settings. However, some of them, along with novel biomarkers, will likely move into wider clinical practice in the coming years.

Functional Consequences of Major or Mild Neurocognitive Disorder Due to Alzheimer's Disease

The prominence of memory loss can cause significant difficulties relatively early in the course. Social cognition (and thus social functioning) and procedural memory (e.g., dancing, playing musical instruments) may be relatively preserved for extended periods.

Differential Diagnosis

Other neurocognitive disorders. Major and mild NCDs due to other neurodegenerative processes (e.g., Lewy body disease, frontotemporal lobar degeneration) share the insidious onset and gradual decline caused by Alzheimer's disease but have distinctive core features of their own. In major or mild vascular NCD, there is typically history of stroke temporally related to the onset of cognitive impairment, and infarcts or white matter hyperintensities are judged sufficient to account for the clinical picture. However, particularly when there is no clear history of stepwise decline, major or mild vascular NCD can share many clinical features with Alzheimer's disease.

Other concurrent, active neurological or systemic illness. Other neurological or systemic illness should be considered if there is an appropriate temporal relationship and severity to account for the clinical picture. At the mild NCD level, it may be difficult to distinguish an Alzheimer's disease etiology from that of another medical condition (e.g., thyroid disorders, vitamin B_{12} deficiency).

Major depressive disorder. Particularly at the mild NCD level, the differential diagnosis also includes major depression. The presence of depression may be associated with reduced daily functioning and poor concentration that may resemble an NCD, but improvement with treatment of depression may be useful in making the distinction.

Comorbidity

Most individuals with Alzheimer's disease are elderly and have multiple medical conditions that can complicate diagnosis and influence the clinical course. Major or mild NCD due to Alzheimer's disease commonly co-occurs with cerebrovascular disease, which contributes to the clinical picture. When a comorbid condition contributes to the NCD in an individual with Alzheimer's disease, then NCD due to multiple etiologies should be diagnosed.
